# Evaluation of eight commercial Zika virus IgM and IgG serology assays for diagnostics and research

**DOI:** 10.1371/journal.pone.0244601

**Published:** 2021-01-26

**Authors:** Swee Ling Low, Yee Sin Leo, Yee Ling Lai, Sally Lam, Hwee Huang Tan, Judith Chui Ching Wong, Li Kiang Tan, Lee Ching Ng

**Affiliations:** 1 Environmental Health Institute, National Environment Agency, Singapore, Singapore; 2 National Centre for Infectious Disease, Singapore, Singapore; 3 Blood Services Group, Health Sciences Authority, Singapore, Singapore; 4 School of Biological Sciences, Nanyang Technological University, Singapore, Singapore; Instituut voor Tropische Geneeskunde, BELGIUM

## Abstract

Several commercial Zika virus (ZIKV) serology assays have been developed since the recognition of ZIKV outbreaks as a Public Health Emergency of International Concern in 2016. However, test interpretation for ZIKV serology can be challenging due to antibody cross-reactivity with other flaviviruses like dengue virus (DENV). Therefore, we sought to evaluate the performance of eight commercially available ZIKV IgM and IgG assays across three testing platforms, namely, immunochromatographic tests (ICT), ELISAs and immunofluorescence tests (IIFT). The test panel comprised of 278 samples, including acute and convalescent sera or plasma from ZIKV-confirmed, DENV-confirmed, non-ZIKV and non-DENV patients, and residual sera from healthy blood donors. The ZIKV IgM and IgG serology assays yielded higher test sensitivities of 23.5% - 97.1% among ZIKV convalescent samples as compared to 5.6% - 27.8% among ZIKV acute samples; the test specificities were 63.3% - 100% among acute and convalescent DENV, non-DENV samples. Among the ELISAs and IIFTs, the Diapro ZIKV IgM ELISA demonstrated high test sensitivity (96%) and specificity (80%) when tested on early convalescent samples, while the Euroimmun ZIKV IgG ELISA yielded the highest test specificity of 97% - 100% on samples from non-ZIKV patients and healthy blood donors. For rapid ICTs, the LumiQuick IgM rapid ICT yielded low test sensitivity, suggesting its limited utility. We showed that commercial ZIKV IgM and IgG serology assays have differing test performances, with some having moderate to high test sensitivities and specificities when used in a dengue endemic setting, although there were limitations in IgG serology.

## Introduction

Zika virus (ZIKV) is a mosquito-borne virus that belongs to the family *Flaviviridae*, genus *Flavivirus*. The virus was first isolated from a rhesus monkey in the Zika forest of Uganda [1], with only few and sporadic cases previously reported in Africa and Asia [2]. In 2007, a large ZIKV outbreak occurred on Yap Island, Federated States of Micronesia, where the disease was reported to be mild [3]. Subsequently, ZIKV infections received significant global public health attention when they were associated with severe neurological complications such as Guillain-Barré syndrome (GBS) in adults and congenital Zika syndrome (includes microcephaly and other severe birth defects) in neonates, in epidemics in French Polynesia and Brazil, respectively [4–8]. This prompted the World Health Organization (WHO) to declare the ZIKV epidemic as a Public Health Emergency of International Concern (PHIC) in February 2016 [9]. Since then, several countries reported autochthonous transmission of ZIKV [10] including Singapore in August 2016 [11, 12]. As of March 2017, 84 countries reported new introductions or re-introductions of ZIKV following the WHO classification [13].

ZIKV is primarily transmitted by infected *Aedes* species mosquitoes, mainly *Aedes aegypti*, which also transmits dengue virus (DENV). Both viruses share similar geographical distribution, transmission cycles and symptoms of infection [14]. The presence of *Aedes aegypti* and *Aedes albopictus* mosquitoes places Singapore at risk of DENV and ZIKV epidemics, with local transmission of both viruses being reported [11, 15–17]. While DENV is endemic in Singapore and cases are reported throughout the year [15], there was only one recorded ZIKV outbreak in 2016, with 458 reported cases [11, 12]. Following which, sporadic ZIKV cases were reported in 2018 and 2019 [18], suggesting that there could be low levels of transmission in Singapore. The threat of future Zika outbreaks in Singapore remains due to the presence of competent vectors [16, 17] and the low levels of immunity against ZIKV in the population (unpublished data, EHI).

Recommended test guidelines for ZIKV include the use of both molecular and serology methods [19–21]. A definitive ZIKV infection in a symptomatic patient is based on ZIKV RNA detection in whole blood, serum or urine [22–24], while the detection of ZIKV-specific antibodies can support ZIKV diagnosis when tests are carried out after the viremia or viruria stage of illness. ZIKV IgM is usually detectable by Day 4 post infection and persists in the first 12 weeks of infection [22, 25], although persistence beyond 12 weeks has also been observed [21]. ZIKV IgG, which is detected after IgM but persists after infection, can be used as a marker for seroconversion and seroprevalence. The interpretation of ZIKV serology results (both IgM and IgG) may be challenging due to antibody cross-reactivity and non-specific reactivity between ZIKV and other flaviviruses such as DENV [25–29]. In scenarios where cross-reactive antibodies are present, the plaque reduction neutralization test (PRNT) is recommended as a confirmatory test to differentiate antibodies to related viruses [21]. However, the PRNT is labor-intensive and has a low sample throughput, which renders it impractical for use in routine diagnostics.

Several commercial serological assays utilizing different methods have been developed since the recognition of ZIKV as a public health threat. There is a need to assess their performance to identify suitable tests for serological diagnosis or serosurveys of ZIKV infections. In a dengue endemic country like Singapore, where dengue seroprevalence is approximately 50% in the resident population [30, 31], it is also important to evaluate the impact of cross-reactivity of DENV antibodies when using ZIKV serological assays in an endemic area. Therefore, in this study, we evaluated the performance of eight ZIKV serology assays [one ZIKV IgM/IgG rapid immunochromatograhic tests (ICT), four ZIKV IgM ELISAs, one ZIKV IgM immunofluorescence Test (IIFT) and two ZIKV IgG ELISA assays] for their potential use.

## Materials and methods

### Sample panel

The test panel comprised of 278 sera or plasma samples from three categories (Set A, Set B and Set C), as summarized in Table 1. Briefly, Set A consisted of 78 acute sera (≤5 days’ post fever onset) collected from patients suspected of having ZIKV or DENV infections in 2016. The samples were collected at primary healthcare clinics in Singapore and sent to the Environmental Health Institute (EHI) of the National Environment Agency (NEA) for diagnostic testing. The ZIKV and DENV infections were confirmed by real-time ZIKV RT-PCR analysis of urine [25] and the rapid Standard Diagnostics (SD) BIOLINE Dengue Duo Non-structural (NS) 1 Ag/IgM/IgG combo test (Standard Diagnostics, Gyeonggi-do, Republic of Korea), respectively.

**Table 1:** Sample panels used in the study.

Sample panel	Sample characteristics	Tests performed for diagnosis/status^a^	Time to collection after the onset of fever (days)	No. of samples	Diagnostic accuracy index
Acute febrile patients’ samples—Set A (n = 78)					
ZIKV subgroup	Confirmed ZIKV infection	Positive ZIKV real-time RT-PCR of the urine sample	≤5	18	Sensitivity
Non- ZIKV subgroup	Confirmed DENV infection	Positive DENV Non-structural protein 1 (NS1) Ag and IgM and IgG		30	Specificity
	Negative ZIKV, DENV	Negative for above tests		30	Specificity
Convalescent samples—Set B (n = 121)					
ZIKV subgroup	Confirmed ZIKV infection	Positive ZIKV real-time RT-PCR of urine sample at first visit	7–14 (Set B1); 23–34 (Set B2)	27 (Set B1)	Sensitivity
				34 (Set B2)	
Non- ZIKV subgroup	Confirmed DENV infection	Positive DENV Non-structural protein 1 (NS1) Ag test or real-time RT-PCR at first visit		15 (Set B1)	Specificity
				15 (Set B2)	
	Negative DENV	Negative for above DENV tests		15 (Set B1)	Specificity
				15 (Set B2)	
Healthy blood donor—Set C (n = 79)					
ZIKV subgroup	Positive ZIKV	Presence of ZIKV NAb by PRNT	NA	19	Sensitivity
Non- ZIKV subgroup	Positive DENV	Positive Dengue IgG Indirect ELISA and presence of DENV NAb by PRNT		30	Specificity
	Negative ZIKV, DENV	Negative Dengue IgG Indirect ELISA and absence of ZIKV NAb by PRNT		30	Specificity

Dengue virus, DENV; neutralizing antibodies, NAb; plaque reduction neutralization test, PRNT; Zika virus, ZIKV; not applicable, NA.

^a^ Tests were performed on serum or plasma samples unless otherwise stated.

Set B consisted of 121 convalescent plasma samples from patients suspected of having DENV or ZIKV infection in two separate studies conducted at Tan Tock Seng Hospital (TTSH), Singapore [32, 33]. Plasma samples were collected from 2010 to 2012, and from August 2016 to September 2016, for the DENV and ZIKV studies, respectively. ZIKV infections were confirmed by real-time ZIKV RT-PCR analysis of urine [25] while DENV infections were confirmed by real-time DENV RT-PCR analysis or DENV NS-1 Ag ELISA (Bio-Rad Platelia NS1 Ag ELISA, Bio-Rad Laboratories, Marnes-la-Coquette, France). The convalescent plasma samples were further grouped into two categories, samples that were collected between 7 to 14 days’ (Set B1, n = 57) and those that were collected between 23 to 34 days’ (Set B2, n = 64) post onset of fever. Among the dengue confirmed patients (n = 15), six and nine patients were identified as primary and secondary DENV infections respectively, using the previously described laboratory criteria [34]. In addition to the dengue patients, samples from an equal number of non-dengue patients (n = 15) were included for specificity testing.

Lastly, to evaluate the performance of the ZIKV IgG ELISAs for use in serosurveys, 79 residual serum samples obtained from healthy blood donors (Set C) were included. Among these, there were 19 samples with both ZIKV and DENV neutralizing antibodies (NAb), 30 samples with DENV NAb only, and 30 non-ZIKV, non-DENV samples as determined by PRNT (described below). Written informed consent was obtained from the blood donors for use of the residual serum samples for research (DSRB Ref: 2013/00791).

This study was reviewed and approved by the NEA-EHI Institutional Review Board (IRB 019/2016). All samples were anonymized before used.

### Serologic analyses

Acute and convalescent dengue patient samples, convalescent non-dengue patient samples, and dengue negative healthy blood donor samples were tested for ZIKV NAb using the previously described PRNT method [30], all other samples were tested for both DENV and ZIKV NAb. Briefly, for the virus-antibody binding reaction, equal volumes (100 μL) of diluted sera (heat-inactivated and diluted 10-fold from 1:10 to 1:1000) and ZIKV or DENV (800 plaque forming units/mL) were added into 96-well plates and incubated at 37°C for an hour. Fifty microliters of the virus–antibody mixture was added into 24-well plates containing Baby Hamster Kidney (BHK) cells (seeded at 1 × 10^5^ cells/ mL) in triplicates. The plates were incubated overnight at 37°C to allow virus adsorption onto BHK cells and were overlaid with 1% carboxymethyl cellulose (CMC) medium the following day. After 4 days of incubation at 37°C, viral plaques were fixed with 20% formalin and stained using 0.1% naphthol blue solution. The plaques were counted and the 50% end-point plaque reduction neutralization titers (PRNT_50_) were computed using the log probit method as described by Russell *et al*. [35]. Antibody titers were expressed as the reciprocal of the end point dilution. The DENV and ZIKV strains used for the PRNT are listed in S1 Table. Samples with ZIKV PRNT_50_ ≥1:30 to both ZIKV strains and DENV PRNT_50_ ≥1:30 to any of the four serotypes indicated the presence of neutralizing activity against the two viruses respectively. The DENV and ZIKV PRNT results of the sample panels are described in S1 Text.

The commercial kits evaluated in this study are listed in Table 2. The tests were performed and results were interpreted according to the manufacturers’ recommendations. The result outcomes for all ELISAs were positive, equivocal/borderline and negative, except for the InBios ZIKV IgM ELISA which had four result outcome categories: presumptive Zika positive, possible Zika positive, presumptive other flavivirus positive, and negative.

**Table 2:** List of ZIKV IgM and IgG serologic assays evaluated.

Kit name (manufacturer)	Serologic marker	Method
Rapid ICT		
LumiQuick QuickProfile™ Zika virus IgG/IgM antibody combo test (LumiQuick Diagnostics, USA)	IgM and IgG	Rapid ICT
ELISA/IIFT		
Diapro ZIKV IgM or IgG ELISA (Diapro, Diagnostics Bioprobes Srl, Milano, Italy)	IgM or IgG	ELISA
Euroimmun Anti-Zika virus IgM or IgG ELISA (Euroimmun, Medizinische Labordiagnostika AG, Lübeck, Germany)	IgM or IgG	ELISA
InBios ZIKV *Detect*^TM^ IgM Capture ELISA^a^ (InBios International Inc, Seattle, WA)	IgM	ELISA
NovaLisa^®^ Zika virus IgM μ-capture ELISA (NovaTec Immundiagnostica GmbH, Dietzenbach, Germany)	IgM	ELISA
Euroimmun Anti-Zika virus IgM IIFT (Euroimmun, Medizinische Labordiagnostika AG, Lübeck, Germany)	IgM	IIFT

Immunochromatographic tests, ICT; Enzyme-linked immunosorbent assay, ELISA; Indirect immunofluorescence test, IIFT.

^a^ Our study evaluated the performance of the older version. The manufacturer has since provided a new version, InBios ZIKV *Detect*^TM^ 2.0 IgM Capture ELISA.

### Data analysis

Diagnostic accuracy indices were calculated for the ZIKV serology assays [36]. The sensitivity, specificity with 95% Wald confidence intervals (CIs) and the Area under Receiver Operating Characteristics Curve (AUROCC) values with 95% CI were determined. The AUROCC is a numerical value outcome (between 0 and 1) that assesses both the test sensitivity and specificity and could be used to determine and compare the diagnostic accuracy of tests. A perfect diagnostic test has an AUROCC value of 1.0 whereas a non-discriminating test has an area of ≤0.5 [37, 38]. The Chi-square test was used for testing significant differences in ZIKV seropositivity obtained by the ZIKV serology assays in primary and secondary DENV samples. Statistical significance was set at *p* < 0.05. All analyses were performed using SPSS (Version 23, IBM) and R software [39].

## Results

### Performance of ZIKV IgM and IgG tests on acute and convalescent samples from ZIKV-confirmed, DENV-confirmed, and non-ZIKV and non-DENV patients

**Fig 1:**
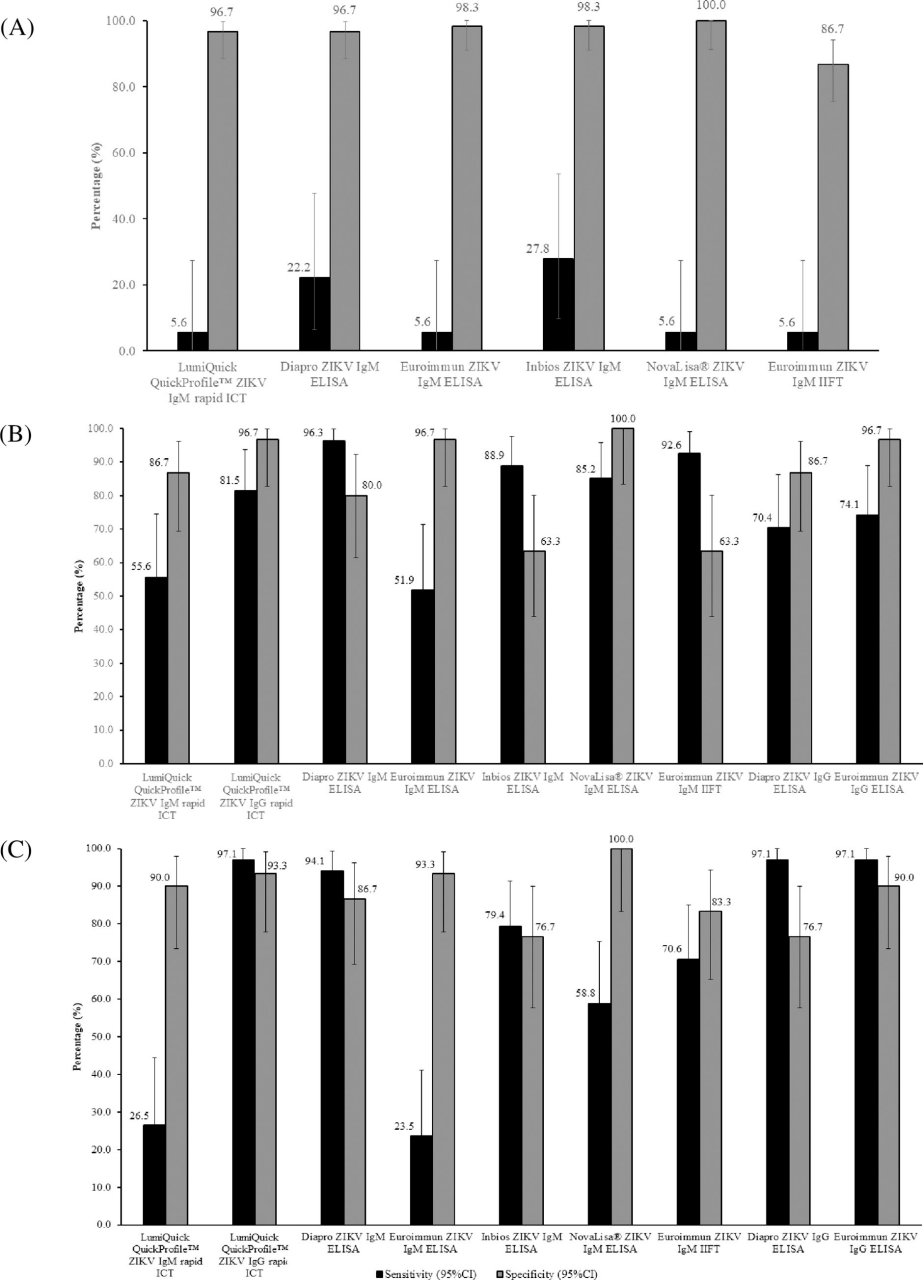
(A) Sensitivity and specificity of ZIKV IgM rapid ICT, ELISAs and IIFT using acute samples from ZIKV-confirmed, DENV-confirmed, and non-ZIKV and non-DENV patients (Set A; n = 78). Sensitivity and specificity of ZIKV IgM/IgG rapid ICT, IgM ELISAs/IIFT and IgG ELISAs using (B) 7–14 days’ (Set B1; n = 57) and (C) 23–34 days’ (Set B2; n = 64) convalescent samples from ZIKV-confirmed, DENV-confirmed, and non-DENV patients. Confidence intervals (black bars) were constructed using Wald’s method.

When acute samples were tested (Set A, n = 78), ZIKV IgM test sensitivities for rapid ICT and ELISA/IIFT tests ranged from 5.6% to 27.8% (Fig 1A), with the InBios ZIKV IgM ELISA yielding the highest ZIKV IgM test sensitivity of 27.8% (95% CI: 9.69%-53.48%) and highest AUROCC value of 0.631 (Table 3). Test specificities for the ZIKV IgM assays ranged from 86.7% to 100% (Fig 1A), with NovaLisa^®^ ZIKV IgM ELISA yielding the highest specificity. The low test sensitivities observed using ZIKV acute samples could be due to the time of collection, whereby antibodies have not been produced <5 days after fever onset. This was further explained by the absence of ZIKV NAb in almost 30% of ZIKV acute samples (S1 Text).

**Table 3:** Test performance characteristics (AUROCC) in acute and convalescent patient samples.

Test/Result	AUROCC (95%CI)		
	Set A—Acute samples (≤5 days’ post fever onset, n = 78)	Set B1 –Convalescent samples (7–14 days’ post fever onset, n = 57)	Set B2 –Convalescent samples (23–34 days’ post fever onset, n = 64)
**ZIKV IgM/IgG ICT Rapid Test**			
LumiQuick QuickProfile™ ZIKV IgM rapid ICT^a^	0.511 (0.356, 0.666)	0.711 (0.573,0.849)	0.582 (0.442, 0.722)
LumiQuick QuickProfile™ ZIKV IgG rapid ICT^a^	NA	0.891 (0.795, 0.987)	0.952 (0.890, 1.000)
**ZIKV IgM ELISA/IIFT**			
**Diapro ZIKV IgM ELISA**	0.594 (0.433, 0.756)	0.881 (0.785, 0.978)	0.904 (0.819, 0.989)
**Euroimmun ZIKV IgM ELISA**	0.519 (0.364, 0.675)	0.743 (0.608, 0.877)	0.584 (0.445, 0.724)
**Inbios ZIKV IgM ELISA**	0.631 (0.468, 0.793)	0.761 (0.633, 0.889)	0.780 (0.662, 0.899)
**NovaLisa® ZIKV IgM ELISA**	0.528 (0.371, 0.685)	0.926 (0.845, 1.000)	0.794 (0.681, 0.907)
**Euroimmun ZIKV IgM IIFT**	0.461 (0.314, 0.608)	0.780 (0.656, 0.904)	0.770 (0.650, 0.889)
**ZIKV IgG ELISA**			
**Diapro ZIKV IgG ELISA**	NA	0.785 (0.660, 0.910)	0.869 (0.770, 0.967)
**Euroimmun ZIKV IgG ELISA**	NA	0.854 (0.745, 0.962)	0.935 (0.864, 1.000)

Area under Receiver Operating Characteristics Curve, AUROCC; not applicable, NA.

^a^ LumiQuick QuickProfile™ ZIKV IgM & IgG rapid ICTs refer to a combo test kit.

**Fig 2:**
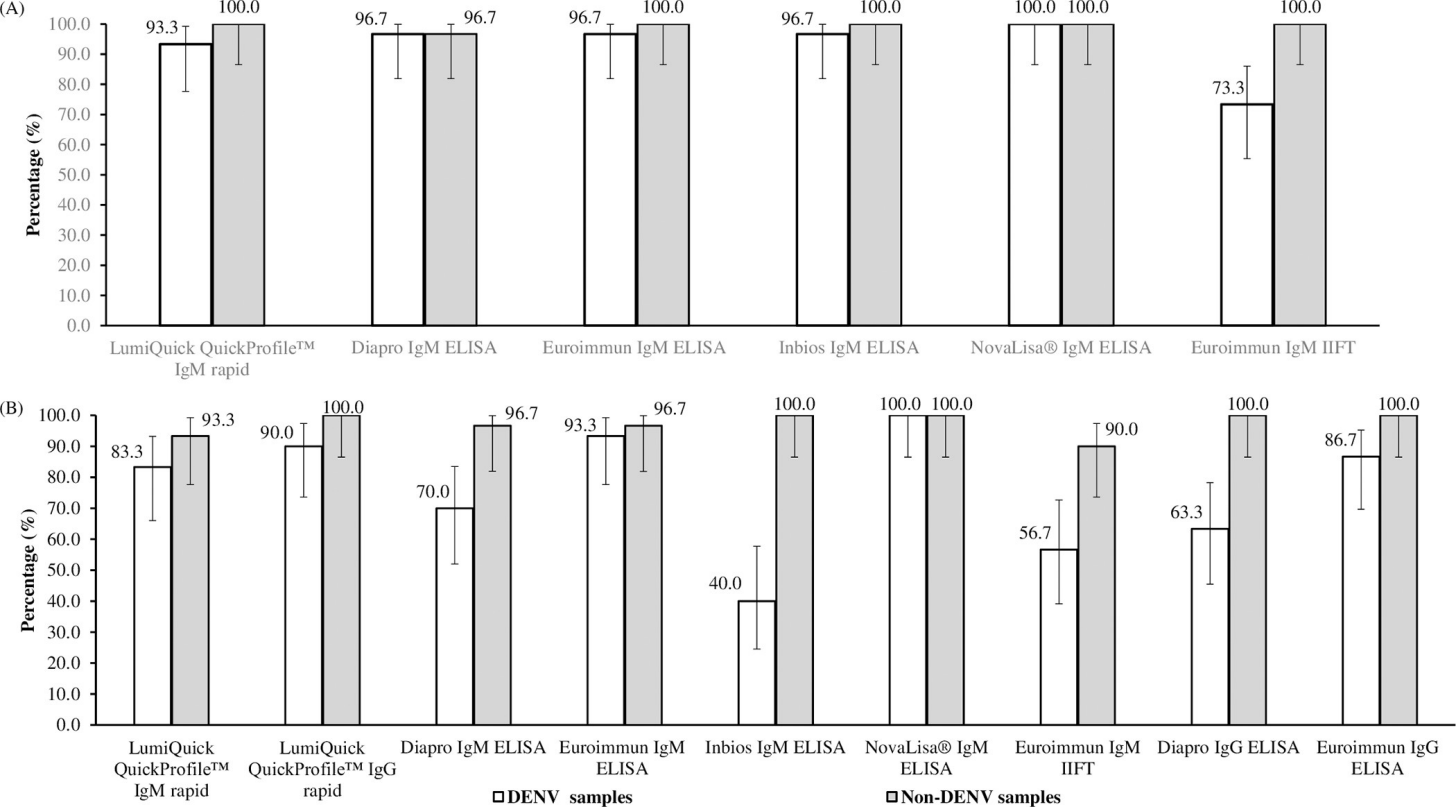
(A) Specificity of ZIKV IgM rapid ICT, ELISAs and IIFT using acute samples from DENV-confirmed (n = 30), and non-ZIKV and non-DENV (n = 30) patients. (B) Specificity of ZIKV IgM/IgG rapid ICT, IgM ELISAs/IIFT and IgG ELISAs using 7–34 days’ convalescent samples from DENV-confirmed (n = 30), and non-DENV patients (n = 30) (Set B). Confidence intervals (black bars) were constructed using Wald’s method. LumiQuick QuickProfile™ ZIKV IgM & IgG rapid ICTs refer to a combo test kit.

For acute DENV and non-DENV, non-ZIKV samples, the test specificities of the ZIKV IgM assays were 73.3% - 100% and 96.7% - 100%, respectively (Fig 2A). The presence of cross-reactive DENV antibodies in the DENV-confirmed samples could have resulted in positive ZIKV results, leading to the lower test specificities. The AUROCC analysis revealed overall low values (AUROCC values of 0.461 to 0.631) for ZIKV IgM tests in acute samples (Table 3).

As expected, test sensitivities improved markedly for ZIKV convalescent samples. Using these samples, the test sensitivities of ZIKV IgM component of rapid ICT and IgM ELISA/IIFT tests ranged from 51.9% to 96.3% for Set B1 (7–14 days’; n = 27) and 23.5% to 94.1% for Set B2 (23–34 days’; n = 34) (Fig 1B and 1C, respectively). Among the tests, the Diapro ZIKV IgM ELISA yielded the highest ZIKV IgM test sensitivity of 94.1% - 96.3%, with a high specificity of 80% - 86.7% and high AUROCC values of 0.881 and 0.904 (Fig 1B and 1C, Table 3).

With regard to ZIKV IgG assays, the test sensitivities for rapid ICT and ELISA ranged from 70.4% to 81.5% in Set B1 and were at 97.1% in Set B2 (Fig 1B and 1C, respectively). For samples collected 7–14 days’ post-fever onset (Set B1), LumiQuick’s ICT yielded the highest ZIKV IgG test sensitivity of 81.5% (95%CI: 61.92%-93.70%) (Fig 1B) and had a high AUROCC value of 0.891 (Table 3), while LumiQuick’s ICT, Diapro’s and Euroimmun’s ELISAs yielded the highest ZIKV IgG test sensitivities of 97.1% (95% CI: 84.67–99.93%) for samples collected 23–34 days’ post-fever onset (Set B2) (Fig 1C).

The test specificities of the ZIKV IgM and IgG tests ranged from 63.3% to 100% in Set B1 (Fig 1B) and 76.7% to 100% in Set B2 (Fig 1C) with NovaLisa^®^ ZIKV IgM ELISA, achieving the highest specificity of 100%. Using the DENV convalescent samples in Set B, ZIKV IgM tests generally achieved higher test specificities as compared to ZIKV IgG tests (IgM: 40% - 100%; IgG: 63.3% - 90%) (Fig 2B). For non-DENV convalescent samples, the test specificities of the ZIKV IgM and IgG tests were 90% - 100% and 100%, respectively (Fig 2B). The AUROCC values of the ZIKV IgM and IgG tests ranged from 0.582 to 0.952 (Table 3). The 2 X 2 contingency table of the ZIKV IgM and IgG tests’ results are available as S2 Table.

### Performance of ZIKV IgG ELISAs on ZIKV and non-ZIKV sera samples from healthy blood donors

The utility of ZIKV IgG ELISAs in ZIKV seroprevalence studies (serosurveys) was further evaluated using residual sera from healthy blood donors that have been tested by PRNT (Set C, n = 79). The test sensitivities of the ZIKV IgG ELISAs ranged from 52.6% to 84.2%, with Diapro ZIKV IgG ELISA yielding the highest sensitivity (Table 4). The test specificities ranged from 90.0% to 100%, with Euroimmun ZIKV IgG ELISA yielding the highest specificity (100%). Among 60 samples that comprised of an equal number of DENV NAb-only samples and DENV/ZIKV NAb-absent samples, Diapro ZIKV IgG ELISA incorrectly identified six of these samples as ZIKV IgG positive, resulting in reduced test specificity (90%). The Euroimmun ZIKV IgG ELISA did not detect any of these samples as ZIKV IgG positive and had 100% specificity.

**Table 4:** Results of ZIKV IgG ELISA using healthy blood donor samples.

Test/Result		Set C—Healthy blood donor samples n = 79			
		ZIKV PRNT status^a^		Sensitivity (95%CI)	Specificity (95%CI)
		Positive	Negative		
Diapro ZIKV IgG ELISA	Positive	16	6	84.2% (60.42, 96.62)	90.0% (79.49, 96.24)
	Equivocal	1	1		
	Negative	2	53		
Euroimmun ZIKV IgG ELISA	Positive	10	0	52.6% (28.86, 75.55)	100% (94.04, 100)
	Negative	9	60		

^a^ ZIKV PRNT positive defined as ZIKV PRNT_50_≥30 to both tested ZIKV strains.

### Comparison of ZIKV seropositivity in primary and secondary DENV convalescent samples

Studies have shown that DENV-positive sera, especially those from secondary DENV infections, may cross-react with ZIKV [27, 28, 40, 41]. In this study, the ZIKV IgM and IgG assays tested 0% - 41.7% of primary DENV samples (n = 12) and 0% - 72.2% of secondary DENV samples (n = 18) to be positive for ZIKV antibodies. Similar to previous reports, a higher proportion of samples were observed to be positive for ZIKV antibodies in secondary DENV convalescent samples (n = 12; χ^2^ = 5.03–7.74, *P*<0.05) as compared to primary DENV samples (n = 18) (Table 5), using the Diapro ZIKV IgM and IgG ELISA, and Euroimmun ZIKV IgM IIFT assays. Conversely, NovaLisa^®^ ZIKV IgM ELISA did not detect any of the primary and secondary DENV samples as positives. The serological differentiation between ZIKV and DENV in regions with a high proportion of secondary DENV infections remains challenging and a larger sample size may be required to validate the above observations.

**Table 5:** ZIKV seropositivity in convalescent primary and secondary DENV patient samples.

Test/Result	Primary dengue (n = 12)	Secondary dengue (n = 18)	Chi-sq ꭓ2	*P*-value
ZIKV IgM/IgG rapid ICT				
LumiQuick QuickProfile™ ZIKV IgM rapid ICT^a^	8.3%	22.2%	0.25	0.62
LumiQuick QuickProfile™ ZIKV IgG rapid ICT^a^	0.0%	16.7%	0.76	0.38
ZIKV IgM ELISA				
Diapro ZIKV IgM ELISA	0.0%	50.0%	6.36	0.01*
Euroimmun ZIKV IgM ELISA	0.0%	11.1%	0.2	0.65
Inbios ZIKV IgM ELISA	41.7%	72.2%	1.67	0.2
NovaLisa® ZIKV IgM ELISA	0.0%	0.0%	NA	NA
Euroimmun ZIKV IgM IIFT	8.3%	66.7%	7.74	0.005*
ZIKV IgG ELISA				
Diapro ZIKV IgG ELISA	8.3%	55.6%	5.03	0.02*
Euroimmun ZIKV IgG ELISA	0.0%	22.2%	1.45	0.22

Not applicable, NA.

^a^LumiQuick QuickProfile™ ZIKV IgM & IgG rapid ICTs refer to a combo test kit.

## Discussion

We compared the test performance of one ZIKV IgM/IgG rapid ICT, four ZIKV IgM ELISAs, one ZIKV IgM IIFT and two ZIKV IgG ELISAs using characterized sample panels from a dengue endemic setting. Moreover, the gold standard PRNT was used as a reference in this study to determine the seroconversion in ZIKV convalescent samples collected from ZIKV RT-PCR-confirmed patients, the presence of ZIKV NAb in healthy blood donor samples, and the presence of probable cross-reactive DENV NAb. It is advantageous that PRNT is able to establish the presence of ZIKV antibodies.

The performance of the assays for use in routine testing, high throughput screening, and serosurveys was assessed. For rapid ICTs, the LumiQuick ZIKV IgM rapid ICT obtained low test sensitivities (5.6% - 55.6%), suggesting its limited utility; there are also limited commercial ZIKV ICTs available. This contrasts with the availability of many robust DENV rapid ICTs that have high sensitivities [42]. Regarding test reliability, the LumiQuick ZIKV rapid ICT initially gave nine invalid test results but was resolved upon retesting.

For high throughput screening, the Diapro ZIKV IgM ELISA yielded test sensitivities of 94.1%– 96.3% and specificities of 80% - 86.7% among ZIKV, DENV and non-DENV convalescent samples. The sensitivity values met the proposed WHO Zika target product profile’s acceptable sensitivity of >95% but the specificity values fell short of the acceptable specificity of >98% for the diagnosis of patients with active infection [43]. The test sensitivity results were consistent with other studies that reported sensitivities of 69% - 100% [44, 45] but test specificities were lower than the reported specificity of 96% [45]. The evaluation data of the ZIKV IgM ELISA/IIFT tests obtained from this study supplemented the limited reports on test specificities in dengue endemic settings and supported the test performance results in prior studies. For instance, the Euroimmun ZIKV IgM ELISA yielded high test specificities of 93.3% - 96.7% but lower test sensitivities of 23.5% - 51.9%, this corroborating with the results from other studies (test specificity >90%; test sensitivity 19.7% - 58.8%) [46–48]. For the NovaLisa^®^ ZIKV IgM ELISA, higher test specificities were reported by Basile *et al*. (98%, n = 155) [49] and for this study (100%, n = 120) as compared to results reported by Safronetz *et al*. [50]. These authors reported reduced test specificities of 66% in negative samples (n = 25) and 70% in DENV-positive samples (n = 10); the difference in performance could be due to the fewer number of samples used.

The use of a specific ZIKV IgG assay for serosurveys can greatly reduce the false positivity rate in a dengue endemic setting. Although this study demonstrated that the ZIKV IgG tests generally achieved lower test specificities compared to ZIKV IgM tests (IgG: 76.7% - 96.7%; IgM: 63.3% - 100% when DENV and non-DENV convalescent samples were used), the Euroimmun ZIKV IgG ELISA, however, exhibited high test specificities in this and other studies (90.0% - 96.7%, and 100%, respectively) [46, 51]. Furthermore, our results showed that its test specificity was 87% when DENV convalescent patient samples were used, and 100% when both the primary DENV patient samples and DENV NAb-positive healthy blood donor samples were used. Notably, the Euroimmun ZIKV IgG ELISA has been used in ZIKV serosurveys conducted in Laos, Martinique, Bolivia and Taiwan [52–55].

A key focus of the study was the assessment of test specificities for the ZIKV IgM and IgG assays using DENV-confirmed patient samples. Reduced specificities of the ZIKV IgM and IgG tests were observed among DENV convalescent samples, likely due to the increase in cross-reactive DENV antibodies in the DENV convalescent samples. Notably, the basal force of infection in Singapore is approximately 1% and dengue infections (and in turn, our samples) could be primary infections [31]. Although other studies and our results suggest higher cross-reactivity among samples from secondary infections [27, 28, 40, 41], the observation of cross-reactivity among DENV samples from primary infections as well emphasizes the need for specific, yet sensitive assays. In this regard, our study distinguished different test specificities among the commercial ZIKV IgM and IgG assays and provided further information on the assays’ performance.

This study highlighted two key challenges of ZIKV serology testing. Firstly, seropositivity of the ZIKV serologic assays was observed in some DENV convalescent samples, due to the presence of cross-reactive DENV antibodies. This finding corroborated with other studies [41, 56] where an in-house ZIKV NS1 Blockade of Binding assay found 17 of 86 secondary DENV samples obtained from Nicaraguan positive for ZIKV [41]. In another study, the analyses of DENV-immune samples collected less than 12 weeks post infection revealed IgG cross-reactivity with the ZIKV-Envelope Domain I and III antigens, although the level of cross-reactivity decreased in samples collected after more than 12 weeks post infection [56]. The use of PRNT could offer a partial solution to the serological differentiation of DENV and ZIKV infections. Similar to other studies [3, 25, 57, 58], specific ZIKV NAb responses were detected in primary ZIKV infections, but extensive cross-reactive ZIKV and DENV NAb responses were detected in secondary ZIKV infections.

Secondly, low ZIKV antibody levels may also complicate the interpretation of serological test results [59]. Interestingly, negative results were consistently obtained using the ZIKV IgM and IgG tests for convalescent samples of one ZIKV-confirmed patient (RT-PCR positive). The ZIKV NAb titres (PRNT_50_ titers: 31–43) were lower than those for DENV NAb [PRNT_50_ titers: DENV-1 (140–180); DENV-2 (340–450); DENV-3 (<100) and DENV-4 (<100)] for convalescent samples from the patient, although the acute sample tested negative for DENV NS1, IgM and IgG, therefore ruling out a recent DENV infection. Therefore, laboratory diagnoses should always be interpreted with all available test results, and correlated with clinical findings.

In this study, test specificities of the recombinant ZIKV NS1-based Euroimmun ZIKV ELISAs, Diapro ZIKV ELISAs, and LumiQuick ZIKV IgM/IgG rapid ICT were higher than that of the InBios ZIKV IgM ELISA, which utilizes a recombinant ZIKV envelope glycoprotein as the antigen. The difference in performance of the ZIKV serology tests could be attributed to the ZIKV antigen used in detection. Antibodies to NS1 antigens appear to be more virus-specific when compared to envelope glycoprotein antigens, likely due to the electrostatic differences in the NS1 surface loop among flaviviruses such as DENV, ZIKV and West Nile virus [29, 60, 61]. Tests that utilizes ZIKV-infected cells such as the Euroimmun ZIKV IgM IIFT also had lower test specificities. This observation corresponds with previous reports that antigen preparations from whole viruses yielded high level of antibody cross-reactivity between ZIKV and DENV [3, 25, 62]. Of the remaining tests, the NovaLisa^®^ ELISA did not specify the ZIKV antigen which is used. In general, if ZIKV serology tests are used in an area where there is no known DENV transmission, tests with high sensitivities would be preferred, and follow-up tests using a more specific method could be done to confirm the results. On the contrary, ZIKV serology tests with high specificities would be preferred if they are performed in areas with known DENV transmission.

This study evaluated a considerable number of ZIKV serologic assays and provided invaluable information of their test performance in a dengue endemic setting.

## Supporting information

S1 Table DENV and ZIKV strains used in PRNT. (DOCX)

S2 Table 2 x 2 contingency table for ZIKV IgM and IgG tests on acute and convalescent ZIKV and non-ZIKV samples. (DOCX)

S1 Text DENV and ZIKV PRNT results. (DOCX)
