# Supplementary material for: Evaluation of eight commercial Zika virus IgM and IgG serology assays for diagnostics and research
Source: PLoS One. 2021 Jan 26;16(1):e0244601. doi: 10.1371/journal.pone.0244601 (PMC7837473; doi:10.1371/journal.pone.0244601)
Supplement: S1 Table — (DOCX) [file pone.0244601.s001.docx]

S1 Table. DENV and ZIKV strains used in PRNT

| **Virus** | **Strain** | **Genotype** |
| --- | --- | --- |
| DENV-1 | EHI0650Y08  (SG(EHI)DED65008,  Genbank Accession number: GQ357692) | Genotype I |
| DENV-2 | EHI1170Y08  (SG(EHI)DED1171008,  Genbank Accession number: GQ357789) | Asian I |
| DENV-3 | EHI0040Y07  (SG(EHI)D3/0040Y09,  Genbank Accession number: GU370052) | Genotype I |
| DENV-4 | EHI462Y04  (SG(EHI)D4/0462Y04,  Genbank accession number: KP792536) | Genotype I |
| ZIKV | Thailand strain,  NCBI accession No. KF993678 | Asian |
| ZIKV | Puerto Rico strain, PRVABC59, ATCC VR-1843  Genbank accession number: KX377337 | Asian |

Dengue virus, DENV; Zika virus, ZIKV; plaque reduction neutralization test, PRNT
