# Supplementary material for: Evaluation of eight commercial Zika virus IgM and IgG serology assays for diagnostics and research
Source: PLoS One. 2021 Jan 26;16(1):e0244601. doi: 10.1371/journal.pone.0244601 (PMC7837473; doi:10.1371/journal.pone.0244601)
Supplement: S2 Table — (DOCX) [file pone.0244601.s002.docx]

S2 Table. 2 x 2 contingency table for ZIKV IgM and IgG tests on acute and convalescent ZIKV and non-ZIKV samples.

| **Test/Result** | | **Set A - Acute samples (≤5 days' post fever onset, n = 78)** | | | | **Set B1 - Convalescent samples (7-14 days' post fever onset, n = 57)** | | | | **Set B2 - Convalescent samples (23-34 days' post fever onset, n = 64)** | | | |
| --- | --- | --- | --- | --- | --- | --- | --- | --- | --- | --- | --- | --- | --- |
|  |  | Zika status | | Sensitivity (95%CI) | Specificity (95%CI) | Zika status | | Sensitivity (95%CI) | Specificity (95%CI) | Zika status | | Sensitivity (95%CI) | Specificity (95%CI) |
|  |  | Positive | Negative |  |  | Positive | Negative |  |  | Positive | Negative |  |  |
| ZIKV IgM/IgG ICT Rapid Tests | | | | | | | | | | | | | |
| LumiQuick QuickProfile™ ZIKV IgM rapid ICT | Positive | 1 | 2 | **5.6%** (0.14, 27.29) | **96.7%** (88.47, 99.59) | 15 | 4 | **55.6%**  (35.33, 74.52) | **86.7%** (69.28, 96.24) | 9 | 3 | **26.5%** (12.88, 44.36) | **90%**  (73.47, 97.89) |
|  | Negative | 17 | 58 |  |  | 12 | 26 |  |  | 25 | 27 |  |  |
| LumiQuick QuickProfile™ ZIKV IgG rapid ICT | Not applicable | | | | | 22 | 1 | **81.5%**  (61.92, 93.70) | **96.7%** (82.78, 99.92) | 33 | 2 | **97.1%** (84.67, 99.93) | **93.3%** (77.93, 99.18) |
|  |  |  |  |  |  | 5 | 29 |  |  | 1 | 28 |  |  |
| ZIKV IgM ELISA/IIFT | | | | | | | | | | | | | |
| Diapro ZIKV IgM ELISA | Positive | 4 | 2 | **22.2%** (6.41, 47.64) | **96.7%** (88.47, 99.59) | 26 | 6 | **96.3%**  (81.03, 99.91) | **80.0%** (61.43, 92.29) | 32 | 4 | **94.1%** (80.32, 99.28) | **86.7%** (69.28, 96.24) |
|  | Equivocal | 2 | 1 |  |  | 0 | 0 |  |  | 0 | 0 |  |  |
|  | Negative | 12 | 57 |  |  | 1 | 24 |  |  | 2 | 26 |  |  |
| Euroimmun ZIKV IgM ELISA | Positive | 1 | 1 | **5.6%**  (0.14, 27.29) | **98.3%** (91.06, 99.96) | 14 | 1 | **51.9%**  (31.95, 71.33) | **96.7%** (82.78, 99.92) | 8 | 2 | **23.5%** (10.75, 41.17) | **93.3%** (77.93, 99.18) |
|  | Borderline | 0 | 0 |  |  | 5 | 0 |  |  | 6 | 0 |  |  |
|  | Negative | 17 | 59 |  |  | 8 | 29 |  |  | 20 | 28 |  |  |
| Inbios ZIKV IgM ELISA | Presumptive Zika positive | 3 | 1 | **27.8%**  (9.69, 53.48) | **98.3%** (91.06, 99.96) | 23 | 0 | **88.9%** (70.84,97.65) | **63.3%** (43.86, 80.07) | 25 | 0 | **79.4%** (62.10, 91.30) | **76.7% (**57.72, 90.07) |
|  | Possible Zika possible | 2 | 0 |  |  | 1 | 11 |  |  | 2 | 7 |  |  |
|  | Presumptive other Flavivirus positive | 0 | 1 |  |  | 0 | 3 |  |  | 0 | 8 |  |  |
|  | Negative | 13 | 58 |  |  | 3 | 16 |  |  | 7 | 15 |  |  |
| NovaLisa® ZIKV IgM ELISA | Positive | 1 | 0 | **5.6%**  (0.14, 27.29) | **100%** (91.20, 100) | 23 | 0 | **85.2%**  (66.27, 95.81) | **100%**  (83.30, 100) | 20 | 0 | **58.8%** (40.70, 75.35) | **100%**  (83.3, 100) |
|  | Equivocal | 0 | 1 |  |  | 1 | 0 |  |  | 0 | 0 |  |  |
|  | Negative | 17 | 59 |  |  | 3 | 30 |  |  | 14 | 30 |  |  |
| Euroimmun ZIKV IgM IIFT | Positive | 1 | 8 | **5.6%**  (0.14, 27.29) | **86.7%** (75.41, 94.06) | 25 | 11 | **92.6%**  (75.71, 99.09) | **63.3%** (43.86, 80.07) | 24 | 5 | **70.6%** (52.52, 84.90) | **83.3%** (65.28, 94.36) |
|  | Equivocal | 2 | 0 |  |  | 0 | 1 |  |  | 1 | 2 |  |  |
|  | Negative | 15 | 52 |  |  | 2 | 18 |  |  | 9 | 23 |  |  |
| ZIKV IgG ELISA | | | | | | | | | | | | | |
| Diapro ZIKV IgG ELISA | Not applicable | | | | | 19 | 4 | **70.4%**  (49.82, 86.25) | **86.7%** (69.28, 96.24) | 33 | 7 | **97.1%** (84.67, 99.93) | **76.7%** (57.72, 90.07) |
|  |  |  |  |  |  | 1 | 0 |  |  | 0 | 1 |  |  |
|  |  |  |  |  |  | 7 | 26 |  |  | 1 | 22 |  |  |
| Euroimmun ZIKV IgG ELISA |  |  |  |  |  | 20 | 1 | **74.1%**  (53.7, 88.9) | **96.7%** (82.78, 99.92) | 33 | 3 | **97.1%** (84.67, 99.93) | **90%**  (73.47, 97.89) |
|  |  |  |  |  |  | 7 | 29 |  |  | 1 | 27 |  |  |
