# Supplementary material for: Evaluation of eight commercial Zika virus IgM and IgG serology assays for diagnostics and research
Source: PLoS One. 2021 Jan 26;16(1):e0244601. doi: 10.1371/journal.pone.0244601 (PMC7837473; doi:10.1371/journal.pone.0244601)
Supplement: S1 Text — (DOCX) [file pone.0244601.s003.docx]

S1 Text. DENV and ZIKV PRNT results of sample panels

The majority of the acute ZIKV samples in Set A had ZIKV neutralizing antibodies (NAb) (72.2%, 13/18), one had DENV NAb and four others did not have both ZIKV and DENV NAb. Among the 13 ZIKV NAb-containing samples, 8 had both ZIKV and DENV NAbs while five had ZIKV NAb positive only (two with ZIKV PRNT_50_ titer ≥30 to the ZIKV Puerto Rico strain only).

Of the 30 DENV acute samples in Set A, 13.3 % (4/30) had ZIKV NAb to both ZIKV strains (PRNT_50_ range: 33 to ≤240), possibly due to the cross-reactive DENV antibodies present. Among the 30 non-ZIKV, non-DENV acute samples, 43.3% (13/30) had DENV NAb only while the remaining samples did not have ZIKV or DENV NAb.

Of the 61 ZIKV convalescent samples, 41% (25/61) had ZIKV NAb only and remaining samples had both ZIKV and DENV NAb. Among the paired convalescent samples from DENV-confirmed patients (n=30), ZIKV NAb was detected in six samples from three patients. DENV-confirmed samples were also presumed to contain DENV NAb because the rapid test indicated the presence of dengue IgG. None of the convalescent samples from non-dengue patients had ZIKV NAb (PRNT_50_≤10).

Among the 79 residual sera from healthy blood donors, 24% (19/79) had both ZIKV and DENV NAb, 38% (30/79) had DENV NAb only and another 38% (30/79) had undetectable DENV and ZIKV NAb.
